# N-Acetylcysteine Protects HPMCs from High-Glucose-Induced Oxidative DNA Damage

**DOI:** 10.3390/antiox15081032

**Published:** 2026-08-19

**Authors:** Tina Oberacker, Tobias Leibold, Adrian Salega, Leonie Kraft, Moritz Schanz, Markus Ketteler, Jörg Latus, Severin Schricker

**Affiliations:** 1 Dr. Margarete Fischer-Bosch Institute for Clinical Pharmacology, Auerbachstr. 112, 70376 Stuttgart, Germany; 2 University of Tübingen, Geschwister-Scholl-Platz, 72074 Tübingen, Germany; 3 Department of General and Visceral Surgery, Robert Bosch Hospital Stuttgart, Auerbachstr. 110, 70376 Stuttgart, Germany; t.leibold@medius-kliniken.de (T.L.); adrian.salega@rbk.de (A.S.); 4 Department of Internal Medicine and Nephrology, Robert Bosch Hospital Stuttgart, Auerbachstr. 110, 70376 Stuttgart, Germany; leonie.kraft@rbk.de (L.K.); moritz.schanz@rbk.de (M.S.); joerg.latus@rbk.de (J.L.); severin.schricker@rbk.de (S.S.); 5 Department of Internal Medicine and Geriatrics, Robert Bosch Hospital Stuttgart, Hohenheimer Str. 21, 70184 Stuttgart, Germany; markus.ketteler@rbk.de

**Keywords:** oxidative stress, thioredoxin-interacting protein, thioredoxin, reactive oxygen species, DNA-damage

## Abstract

Peritoneal dialysis (PD) is an effective renal replacement therapy; however, its long-term use is limited by the detrimental effects of glucose-based PD fluids on the peritoneal membrane, contributing to fibrosis and ultrafiltration failure. Previous studies have demonstrated that high-glucose exposure promotes oxidative DNA damage through upregulation of thioredoxin-interacting protein (TXNIP) expression, resulting in reduced thioredoxin (Trx) activity. This study investigated strategies to reduce oxidative stress in human peritoneal mesothelial cells exposed to high glucose concentrations. TXNIP expression, Trx activity, intracellular oxidative stress levels, and oxidative DNA damage were analyzed. High-glucose exposure caused a dose-dependent increase in TXNIP expression, a 5–15% reduction in Trx activity, and increased intracellular oxidative stress levels and oxidative DNA damage. Pre-treatment with the ROS scavenger N-acetylcysteine (NAC) reduced these effects. These findings demonstrate that glucose-induced TXNIP upregulation disrupts cellular redox homeostasis, resulting in increased intracellular oxidative stress and oxidative damage. Antioxidant compounds may therefore represent promising therapeutic strategies to protect the peritoneal membrane and improve long-term outcomes in PD.

## 1. Introduction

For patients with end-stage renal disease, peritoneal dialysis (PD) is an effective modality of renal replacement therapy (RRT) that also provides a high degree of patient autonomy [1,2]. Nevertheless, long-term PD is limited by the continuous exposure of human peritoneal mesothelial cells (HPMCs) within the peritoneal membrane (PM) to glucose-based PD fluids (PDFs) and glucose degradation products. This exposure induces structural alterations in the PM, including peritoneal fibrosis, ultimately leading to ultrafiltration failure and technique failure [3,4].

At low levels, reactive oxygen species (ROS) are essential for various physiological processes [5,6,7,8]. However, excessive ROS production damages critical cellular components and contributes to the initiation and progression of numerous diseases [9,10], including PM injury [11,12].

Therefore, maintenance of redox homeostasis is crucial for cellular integrity. To counteract oxidative stress (OS), cells have developed sophisticated antioxidant defense mechanisms, including metallothioneins (MTs) [13]; enzymatic antioxidants such as superoxide dismutase (SOD), catalase (CAT), and glutathione peroxidase (GPx) [14]; and the thioredoxin (Trx)/thioredoxin-interacting protein (TXNIP) system [15,16,17,18]. TXNIP expression is known to increase in response to glucose exposure, glucose-based PDFs [19,20,21,22,23], and diabetic conditions [15]. Moreover, TXNIP acts as a negative regulator of Trx by binding to its catalytic active site [16,24], thereby shifting the cellular redox balance toward oxidative damage. We recently elucidated a pathological mechanism demonstrating that glucose-based PDFs promote oxidative DNA damage in PD patients through TXNIP upregulation, resulting in reduced Trx activity [11].

The aim of the present study was therefore to investigate in primary, living HPMCs whether high-glucose exposure mimicking peritoneal dialysis conditions induces TXNIP-mediated impairment of the thioredoxin system, increased ROS formation and oxidative DNA damage. To test whether these effects are driven by ROS, we used N-acetylcysteine (NAC) as a well-characterized proof-of-concept ROS scavenger.

## 2. Materials and Methods

### 2.1. Chemicals

All chemicals were obtained from Sigma-Aldrich (Taufkirchen, Germany) unless stated otherwise.

### 2.2. Ethical Statement

The study was conducted in accordance with the Declaration of Helsinki and approved by the Ethics Committee of the Eberhard Karls University Tübingen, Germany (process number: approval number 317/2019BO1, approval date: 18 September 2019, updated 4 September 2024; approval number 609/2019BO2, approval date: 16 September 2019, updated 8 July 2024). Written informed consent was obtained from all subjects involved in the study.

### 2.3. Primary Human Peritoneal Mesothelial Cells (HPMCs)

HPMCs were isolated from the peritoneal dialysis effluent (PDE) obtained from patients who had peritoneal dialysis catheters placed for less than 2 weeks. At least 500 mL of PDE was centrifuged at 400× *g* for 5 min at room temperature, the supernatant was discarded, and the cell pellet was washed with PBS (5 min, 400× *g*, RT). The supernatant was discarded again, and the cell pellet was resuspended in E199 medium (M199; Gibco, Thermo Fisher Scientific, Darmstadt, Germany), supplemented with 10% (*v*/*v*) fetal calf serum (FCS; Sigma-Aldrich, Taufkirchen, Germany) and antibiotics (100 U/mL penicillin streptomycin; Gibco), and seeded into a T25 cell culture flask and cultured at 37 °C in a humidified atmosphere containing 5% CO_2_. HPMCs were used between passages 2 and 4. HPMCs obtained from individual patients were maintained and studied separately, and no pooling of HPMCs from different donors was performed. Mesothelial cell identity was validated by performing immunocytochemical staining using the following antibodies: anti-calretinin (M724529-2; Dako, Hamburg, Germany; 1:200) and anti-vimentin (347M-14; Medac, Wedel, Germany; 1:200) (see Figure S1). Table 1 provides an overview of the clinical data and descriptive statistical characteristics of the donors.

### 2.4. Treatment Conditions

All cells were incubated for 3 h under three experimental conditions: control medium with normal glucose concentration (5 mM), and two conditions with high-glucose concentrations (1.5% [83.3 mM] and 4.25% [235.8 mM]). These concentrations were chosen to reflect those of clinically used peritoneal dialysis fluids (Fresenius Medical Care, Bad Homburg, Germany). For antioxidant experiments, cells were pre-incubated with 10 mM NAC [25] for 30 min prior to treatment.

### 2.5. Viability Assay

Cell viability after glucose stimulation was assessed using the CellTiter-Glo^®^ 2.0 Assay (Promega, Walldorf, Germany), which quantifies ATP as an indicator of metabolically active cells [26]. Luminescence, which is directly proportional to the number of viable cells, was measured using a multimode microplate reader (VictorNivo, PerkinElmer, Waltham, MA, USA). Results were normalized to control conditions and are expressed as percentage viability relative to control medium.

### 2.6. Apoptosis Assay

Apoptosis was assessed by measuring caspase-3/7 activity using the Caspase-Glo^®^ 3/7 Assay (Promega, Walldorf, Germany) according to the manufacturer’s instructions. The assay quantifies the activity of the executioner caspases 3 and 7 by detecting luminescence generated upon cleavage of a DEVD-containing luminogenic substrate. Luminescence was measured using a multimode microplate reader (VictorNivo, PerkinElmer, Waltham, MA, USA). Caspase-3/7 activity was normalized to the control condition and is expressed as a percentage of the control.

### 2.7. Cell Lysis

Cells were lysed by adding 75 µL of ice-cold TRX lysis buffer (20 mM HEPES, pH 7.9; 100 mM KCl; 300 mM NaCl; 10 mM EDTA; 0.1% [*v*/*v*] Triton X-100) per 7 × 10^5^ cells. Protease Inhibitor Cocktail III (Roche, Grenzach-Wyhlen, Germany) was added at 1:100 (*v*/*v*) immediately prior to use, and samples were incubated on ice for 30 min. Lysates were then centrifuged at 13,300 rpm at 4 °C for 30 min. Supernatants were collected, and protein concentration was determined using a BCA assay according to the manufacturer’s instructions.

### 2.8. Thioredoxin Activity Assay

Thioredoxin (Trx) activity was determined using the Thioredoxin Fluorescent Activity Assay Kit (Cayman Chemical, Ann Arbor, MI, USA) according to the manufacturer’s instructions. Briefly, 20 µg of total protein was used for each sample. The assay is based on the Trx-dependent reduction in eosin-labeled insulin disulfides, resulting in the release of eosin and a proportional increase in fluorescence. Under conditions in which Trx activity is rate-limiting, the increase in fluorescence is directly proportional to the Trx activity in the sample [27]. Fluorescence was measured at an excitation wavelength of 520 nm and an emission wavelength of 545–560 nm using a multimode microplate reader (VictorNivo, PerkinElmer, Waltham, MA, USA). Results are expressed as percentage TRX activity relative to control conditions.

### 2.9. Immunofluorescence of Cells/Detection of Intracellular Oxidative Stress

For immunofluorescence analysis, sterile coverslips (Roth, Karlsruhe, Germany) were placed in 12-well cell culture plates. A total of 3 × 10^5^ cells per coverslip were seeded and allowed to adhere and grow overnight at 37 °C in a humidified atmosphere containing 5% CO_2_.

CellROX Green (5 µM; Thermo Fisher Scientific, Dreieich, Germany) was added for an additional 30 min at 37 °C and 5% CO_2_ to detect intracellular oxidative stress. The cells were then washed once with PBS and fixed with 4% formalin for 10 min at room temperature (RT). After an additional PBS wash, coverslips were mounted using ProLong™ Glass Antifade Mountant with DAPI (Thermo Fisher Scientific, Dreieich, Germany) and stored at 4 °C until analysis.

For TXNIP expression and oxidative DNA damage analysis, cells were processed as described above. Following fixation, cells were permeabilized with 0.1% (*v*/*v*) Triton X-100 in PBS for 10 min at RT and blocked with 1% (*v*/*v*) bovine serum albumin (BSA) in PBS for 1 h at RT. Cells were then incubated overnight at 4 °C with primary antibodies diluted in blocking buffer: anti-TXNIP (clone JY2, MBL, Tokyo, Japan; 1:500) and anti-γH2AX (clone 20E3, Cell Signaling Technology, Danvers, MA, USA; 1:400).

The following day, coverslips were washed three times with PBS and incubated with the appropriate fluorophore-conjugated secondary antibodies (1:1000 in blocking buffer; anti-rabbit Alexa Fluor 488 and anti-mouse Alexa Fluor 647) for 1 h at RT in the dark. Finally, coverslips were washed three times with PBS, mounted with DAPI-containing mounting medium, and stored at 4 °C until imaging.

Immunofluorescence staining was analyzed using confocal laser scanning microscopy (TCS SP8/DMI6000, Leica Microsystems, Wetzlar, Germany). Mean fluorescence intensity was quantified using ImageJ (v1.52v) and normalized to control conditions, and nuclear foci were quantified using ImageJ (v1.52v). The number of foci per nucleus was determined using the Analyze Particles function. Cells with overlapping nuclei, incomplete nuclei at the image borders, or clear segmentation artifacts were excluded from the analysis. The average number of nuclear foci per nucleus was calculated for each replicate and, for each experimental condition, at least three independent biological replicates were analyzed.

### 2.10. Quantitative PCR (qPCR)

RNA from 8 × 10^5^ cells was extracted using the RNeasy Mini Kit (Qiagen, Hilden, Germany) according to the manufacturer’s instructions. A total of 1 µg of RNA was reverse-transcribed using an RT-PCR kit (Applied Biosystems, Darmstadt, Germany) and subsequently diluted 1:1 with nuclease-free water. Quantitative PCR (qPCR) was performed using Power SYBR Green PCR Master Mix (Applied Biosystems, Darmstadt, Germany), and gene expression was analyzed on a 7500 Real-Time PCR System using Sequence Detection Software (v2.3; Applied Biosystems, Darmstadt, Germany). Relative gene expression was calculated using the ΔΔCt method, with GAPDH as the reference gene.

The following primer sequences were used: GAPDH (forward: 5′-GCA TCT TCT TTT GCG TCG-3′; reverse: 5′-TGT AAA CCA TGT AGT TGA GGT-3′), TXNIP (forward: 5′-AGA CCA GCC AAC AGG TGA GA-3′; reverse: 5′-TGA AGG ATG TTC CCA GAG GC-3′), TRX (forward: 5′-GAC GCT GCA GGT GAT AAA-3′; reverse: 5′-CTG ACA GTC ATC CAC ATC TAC-3′), SOD1 (forward: 5′-CAA TGT GAC TGC TGA CAA AG-3′; reverse: 5′-GTG CGG CCA ATG ATG CAA T-3′), SOD2 (forward: 5′-GAC AAA CCT CAG CCC TAA CG-3′; reverse: 5′-GAA ACC AAG CCA ACC CCA AC-3′), and catalase (forward: 5′-GCC TGG GAC CCA ATT ATC TT-3′; reverse: 5′-GAA TCT CCG CAC TTC TCC AG-3′).

### 2.11. Statistics

Statistical analyses were descriptive only. Continuous clinical variables are presented as median and interquartile range (IQR). Data obtained from primary HPMCs of three independent donors are shown individually to illustrate biological variability between donors. Owing to the limited number of biological replicates, no inferential statistical analyses, normality testing, or multiple-comparison adjustments were performed.

## 3. Results

In a previous study, we demonstrated that glucose-based PD fluids contribute to oxidative DNA damage in peritoneal dialysis patients by inducing TXNIP expression and thereby reducing Trx activity [11].

To investigate therapeutic strategies for mitigating oxidative stress and oxidative DNA damage, we isolated primary human peritoneal mesothelial cells from peritoneal dialysis effluent samples obtained from patients enrolled in our large peritoneal dialysis biobank.

The study population consisted of 10 donors, including four females and six males, reflecting a relatively balanced sex distribution. The median age of the donors was 67 years (IQR: 55.5–68.5), and their median BMI was 25.6 (21.8–32.3) kg/m^2^. Among them, 20% were diagnosed with diabetes mellitus, while hypertension was present in 90% of the donors. Regarding smoking status, 20% of donors were active smokers. Evaluation of routine laboratory parameters revealed no marked alterations among study participants, aside from elevated creatinine and urea concentrations. Leukocyte counts, hemoglobin levels, and CrP values were not indicative of relevant pathological findings. The baseline characteristics of the cohort are presented in Table 1.

### 3.1. Morphological Observations and Cell Viability

**Figure 1:**
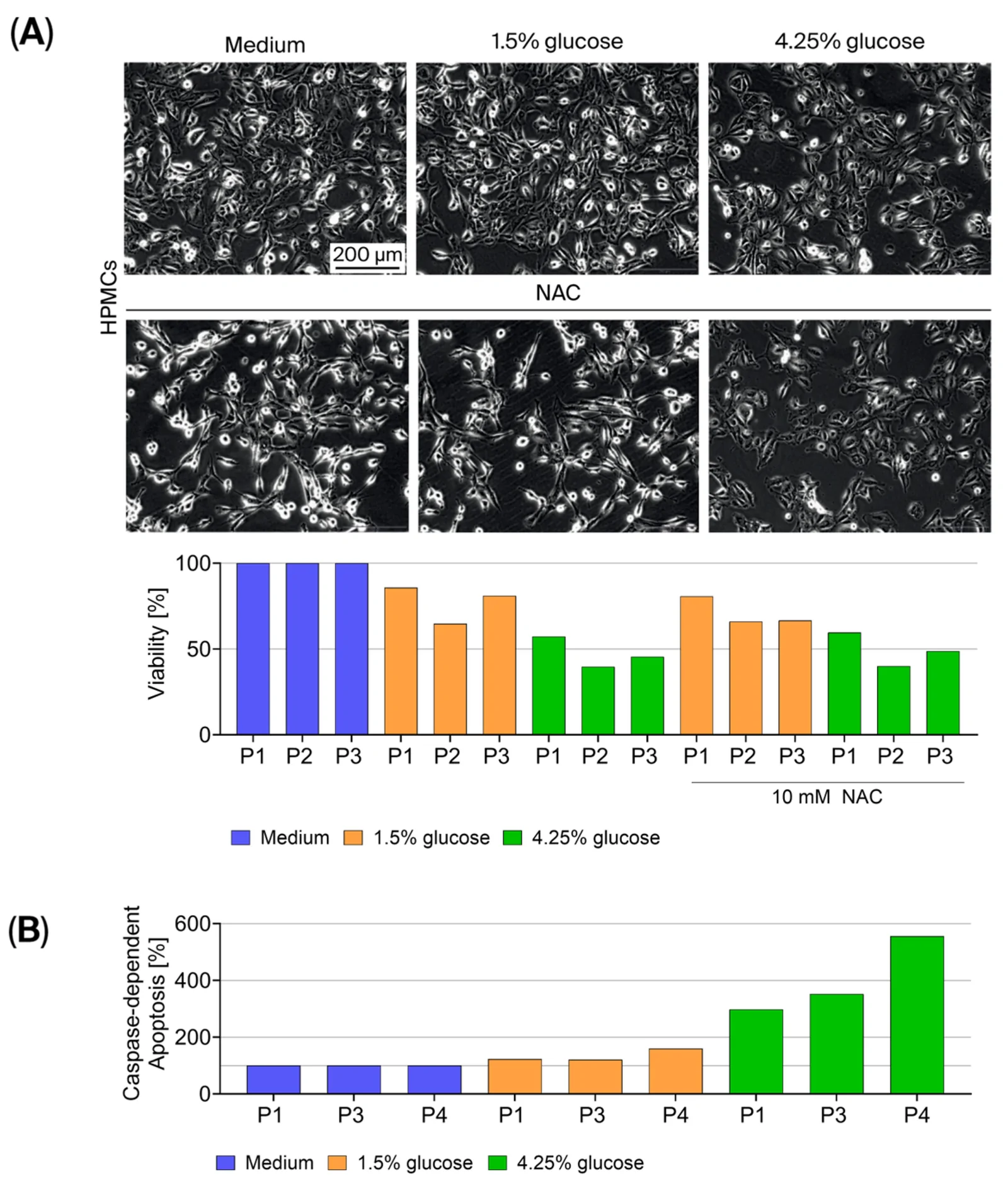
High-glucose exposure reduces viability and induces caspase-dependent apoptosis in primary human peritoneal mesothelial cells (HPMCs). (**A**) Cell morphology was evaluated by phase-contrast microscopy after incubation in medium, or high-glucose medium, in the presence or absence of N-acetylcysteine (NAC) for 3 h. Representative images are shown. Scale bar: 200 µm. Cell viability was assessed by intracellular ATP quantification in HPMCs isolated from three independent patients and normalized to the medium control (100%). (**B**) Apoptosis was assessed by measuring caspase-3/7 activity using the Caspase-Glo^®^ 3/7 Assay. High-glucose exposure significantly increased this activity compared with the medium control, indicating activation of caspase-dependent apoptosis. Data represent cells derived from three independent patients, normalized to the medium control (100%).

Cellular morphology was assessed following exposure to high-glucose medium in the presence or absence of NAC, and it was found that HPMCs exhibited only minimal morphological changes following high-glucose exposure, regardless of NAC treatment (Figure 1A). Cell viability was subsequently evaluated using the CellTiter-Glo assay, whereas high-glucose treatment was associated with a reduction in cell viability compared with the medium control, with approximately 50% of the cells remaining viable after 3 h of incubation under high-glucose conditions (Figure 1A).

To further characterize the reduction in cell viability, apoptosis was assessed by measuring caspase-3/7 activity. High-glucose exposure increased this activity compared with the medium control, with increased activation under high-glucose conditions (Figure 1B). These findings are consistent with involvement of caspase-dependent apoptotic processes in the observed reduction in cell viability.

### 3.2. Upregulation of TXNIP in Response to High-Glucose Treatment

**Figure 2:**
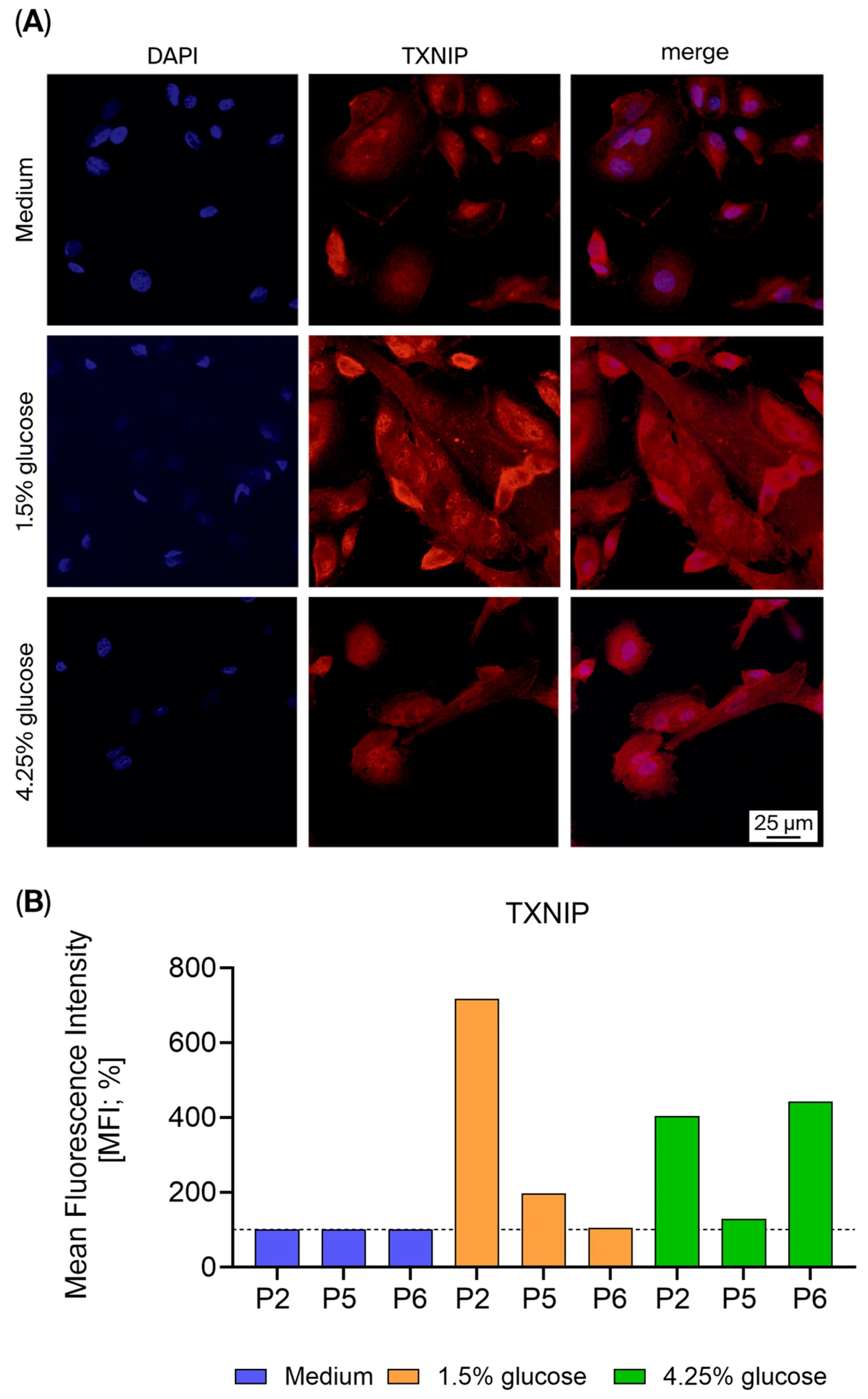
Increased TXNIP expression upon high-glucose exposure in HPMCs. (**A**) Representative confocal microscopy images of primary human peritoneal mesothelial cells (HPMCs) treated with control medium, or high-glucose medium. TXNIP expression was visualized by immunofluorescence using specific antibodies. TXNIP is shown in red, nuclei are stained with DAPI (blue), and merged images are shown in pink. Scale bar: 25 µm. (**B**) Bar graphs represent the mean fluorescence intensity (MFI) obtained from HPMCs derived from three independent patients. For each condition, fluorescence intensity was quantified in 10 nuclei and normalized to the medium control (set to 100%). TXNIP: Thioredoxin-interacting protein.

It has been known for several years that high concentrations of glucose induce TXNIP expression in a human primary peritoneal cell line [22]. Immunofluorescence staining was used to confirm the TXNIP expression in the primary mesothelial cells isolated from our patients and to determine whether such expression could likewise be induced by glucose. Representative images demonstrate basal TXNIP expression in primary HPMCs and an increase following high-glucose stimulation (Figure 2A). To further evaluate these observations, this expression was quantified semi-quantitatively by measuring the mean fluorescence intensity (MFI). Quantification was performed in HPMCs derived from three independent patients and showed higher TXNIP expression following high-glucose exposure (Figure 2B).

To additionally explore whether high-glucose exposure was associated with changes in TXNIP and thioredoxin (TRX) mRNA expression levels, exploratory quantitative real-time PCR analyses were performed in samples from one patient. These preliminary data did not show clear changes in TXNIP or TRX mRNA expression levels following high-glucose exposure and are therefore presented in the Supplementary Materials (Figure S2). Given the single-patient sample size, these findings were not subjected to statistical analysis and cannot be used to draw conclusions regarding transcriptional regulation.

### 3.3. Concurrent Downregulation of Trx Activity and Antioxidant Enzyme Expression Disrupts Cellular Redox Balance

**Figure 3:**
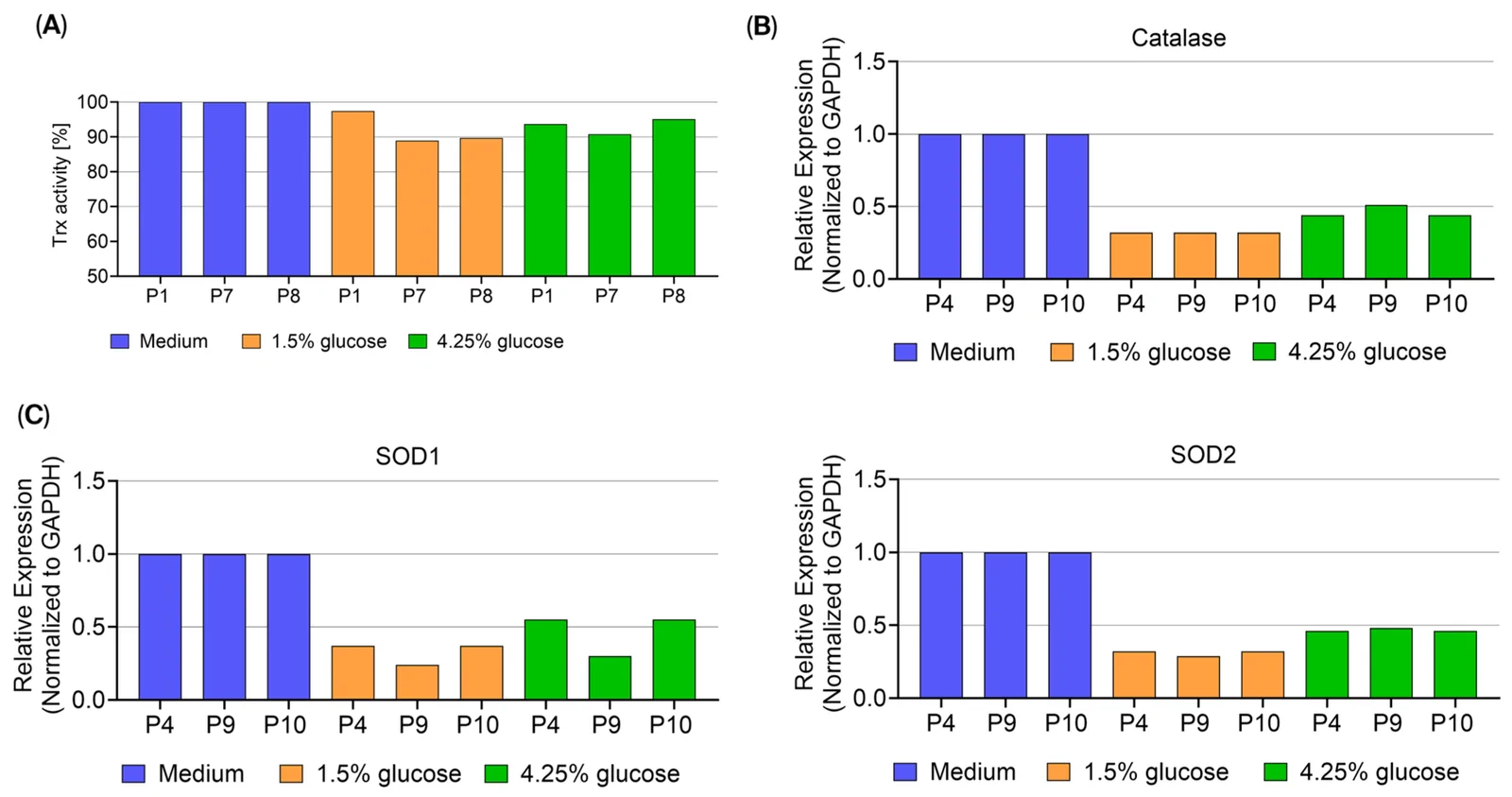
Reduced thioredoxin activity and mRNA expression levels of redox-related genes following high-glucose exposure. (**A**) Trx activity was measured in protein lysates from three independent primary cell isolations and normalized to the medium control (100%). (**B**,**C**) Expression levels of redox-related genes, including catalase (**B**) and superoxide dismutase 1 and 2 (SOD1 and SOD2; (**C**)), were assessed by qPCR. Bars depict results from three independent donors.

TXNIP is a well-established negative regulator of Trx [17]. Therefore, reduced Trx activity may contribute to alterations in cellular redox homeostasis and a more pro-oxidative cellular state. To assess this, Trx activity was measured in protein lysates from primary cells derived from three independent patients following exposure to high-glucose medium. Compared with the medium control, high-glucose exposure resulted in a 5–15% reduction in Trx activity (Figure 3A). Furthermore, we examined the expression levels of key redox-related genes, including catalase and SODs. We observed downregulations of SOD1, SOD2 and catalase mRNA expression following glucose treatment (Figure 3B,C). These changes in antioxidant-related gene expression are consistent with altered redox homeostasis under high-glucose conditions.

**Figure 4:**
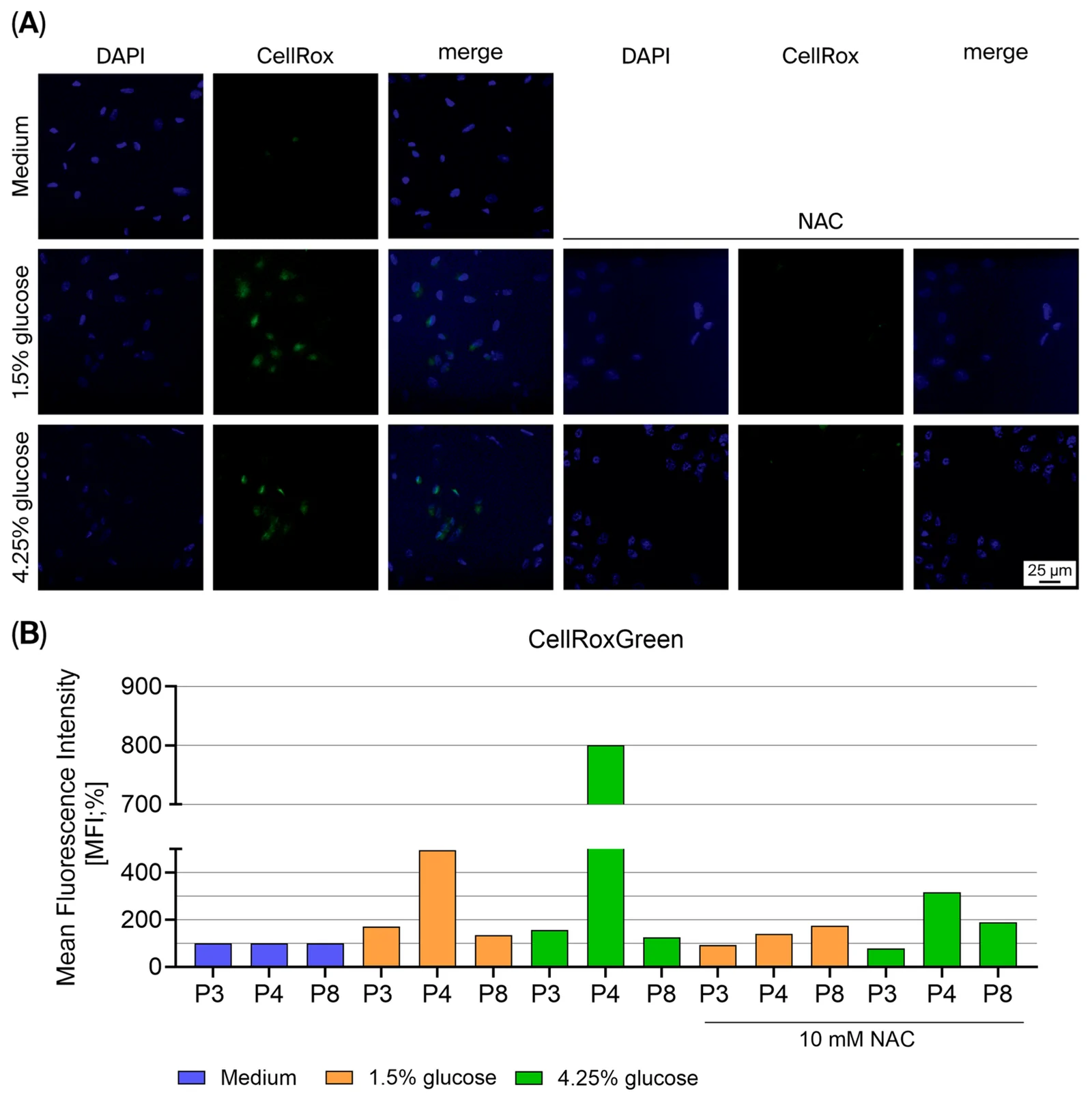
High-glucose-induced intracellular oxidative stress is attenuated by NAC treatment. (**A**) Representative confocal microscopy images of primary cells treated with control medium, or high-glucose medium, in the presence or absence of NAC for 3 h. Intracellular oxidative stress was assessed using the redox-sensitive dye CellROX Green. CellROX Green fluorescence is shown in green, nuclei are counterstained with DAPI (blue), and merged images are shown in turquoise. Scale bar: 25 µm. (**B**) Bar graphs show MFI from three independent donors. For each donor, 10 nuclei were analyzed, and values were normalized to the control medium (set to 100%). MFI: mean fluorescence intensity, NAC: N-acetylcysteine.

The reduction in Trx activity was accompanied by an increase in the CellRoxGreen fluorescence signal. Intracellular oxidative stress was assessed by measuring the mean fluorescence intensity (MFI) of the redox-sensitive dye CellROX Green. High-glucose exposure increased CellROX Green fluorescence in primary HPMCs compared with the medium control, consistent with increased intracellular oxidative stress (Figure 4A,B). Treatment with NAC, an antioxidant that modulates cellular redox homeostasis, reduced the glucose-induced CellROX Green fluorescence signal (Figure 4A,B), consistent with attenuation of oxidative stress response.

### 3.4. Increased Intracellular Oxidative Stress Is Associated with Enhanced Oxidative DNA Damage

**Figure 5:**
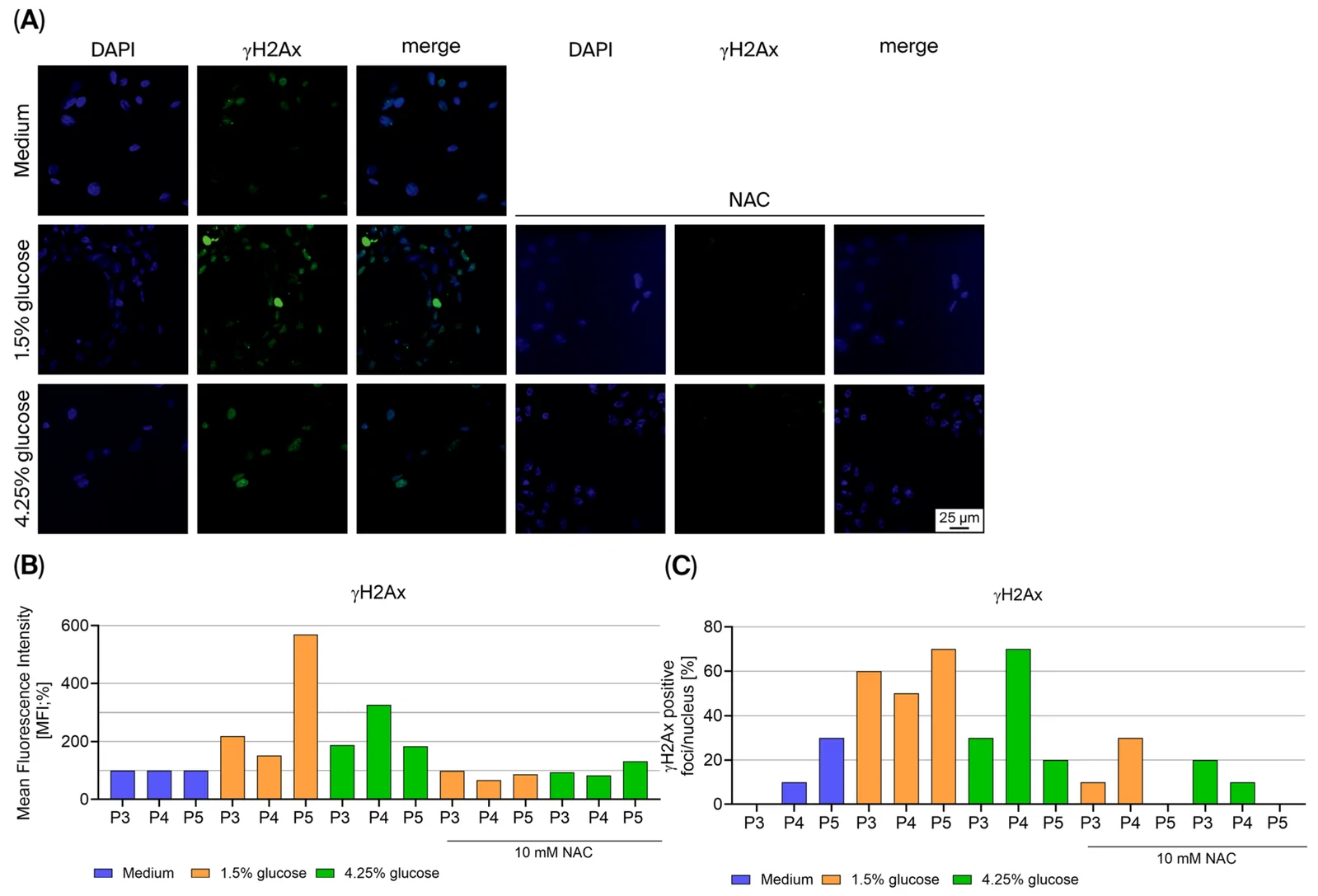
Oxidative DNA damage is attenuated by NAC treatment. (**A**) Representative confocal microscopy images of HPMCs treated with control medium, or high-glucose medium, in the presence or absence of NAC. Oxidative DNA damage was assessed by immunofluorescence staining using anti-γH2Ax antibodies. γH2Ax is shown in green, nuclei are stained with DAPI (blue), and merged images are shown accordingly. Scale bar: 25 µm. (**B**) Bar graphs show MFI from three independent patients. For each patient, 10 nuclei were analyzed, and values were normalized to the medium control (set to 100%). (**C**) Bar graphs show γH2ax-positive foci from three independent patients. For each patient, 10 nuclei were analyzed, and values were normalized to the medium control (set to 100%). MFI: mean fluorescence intensity, NAC: N-acetylcysteine.

Finally, DNA damage was assessed as a downstream cellular response associated with increased oxidative stress. DNA damage was evaluated by immunofluorescence staining of γH2Ax, a well-established marker of DNA double-strand breaks [28,29]. Representative confocal microscopy images revealed an increase in γH2Ax staining following high-glucose exposure (Figure 5A). Semi-quantitative analysis based on both mean fluorescence intensity (MFI) and the number of nuclear foci showed higher γH2Ax levels and an increased number of nuclear foci in high-glucose-treated cells compared with the medium control (Figure 5B,C). Pre-incubation with NAC reduced the high-glucose-induced γH2Ax signal, as reflected by a reduction in both MFI and the number of nuclear foci (Figure 5A–C). These findings are consistent with an involvement of oxidative stress in DNA damage response induced by high-glucose exposure.

In the present study, we investigated the involvement of TXNIP in glucose-induced oxidative stress and DNA damage. High-glucose stimulation increased TXNIP expression in primary HPMCs, as determined by immunofluorescence analysis, and was accompanied by a significant reduction in Trx activity. Consistent with altered redox regulation, high-glucose exposure resulted in increased intracellular oxidative stress, as assessed by CellROX Green staining. Treatment with NAC reduced the CellROX Green fluorescence signal. Furthermore, DNA damage was assessed by immunofluorescence staining for phosphorylated histone H2Ax. High-glucose stimulation increased γH2Ax levels, whereas NAC treatment reduced γH2Ax accumulation. Together, these findings indicate that high-glucose exposure is associated with TXNIP upregulation, reduced Trx activity, increased intracellular oxidative stress, and increased γH2Ax-associated DNA damage in primary HPMCs. The attenuations of oxidative stress-associated signal and γH2Ax accumulation following NAC treatment further supports an involvement of oxidative stress in the cellular response to high-glucose exposure.

## 4. Discussion

Long-term exposure to glucose-based dialysate is known to induce pathophysiological changes in the peritoneal membrane (PM), including peritoneal fibrosis, ultrafiltration failure, and mesothelial cell loss [11,22,30].

Here, we demonstrate that primary peritoneal mesothelial cells exhibit only minimal morphological changes following glucose treatment. However, the cells appear to tolerate the high-glucose conditions and do not show the expected rounded morphology that might be anticipated under increased osmotic stress. The viability assay further indicates that approximately 50% of the cells remain viable after treatment. Furthermore, we confirmed that high-glucose exposure induces TXNIP expression in HPMCs, in agreement with the findings of Wu and colleagues [22]. Notably, we show that TXNIP induction leads to a reduction in the activity of its negative regulator, Trx. The observed alteration in enzyme activity is known to disrupt redox homeostasis, thereby enhancing oxidative stress and oxidative damage as well. Furthermore, we observed increased levels of ROS and the resulting oxidative DNA damage following high-glucose treatment. Hung and colleagues also demonstrated that high glucose concentrations in dialysis fluids induce oxidative stress and apoptosis in HPMCs [31], an effect that could be partially rescued by the ROS scavenger NAC. While a modest reduction in Trx activity may primarily affect redox signaling, the observed downregulations of SOD1, SOD2, and catalase mRNA suggests a reduced antioxidant defense capacity that may contribute to impaired ROS detoxification. However, since these enzymes were assessed only at the transcriptional level, further studies are required to determine whether these changes translate into alterations in protein abundance and enzymatic activity. This study confirms our findings from the ex vivo study investigating the effects of peritoneal dialysis on TXNIP expression and its impact on redox homeostasis in human peritoneal biopsy samples [11].

In general, maintaining redox homeostasis is critical for cellular viability, activation, proliferation and organ function [32,33]. Furthermore, the pathogenesis of chronic kidney disease (CKD) and peritoneal dialysis-associated membrane alterations is associated with increased ROS accumulation and oxidative damage [11,34,35,36,37,38,39].

Therefore, strategies aimed at reducing oxidative stress, including antioxidant approaches, are of considerable interest. Accordingly, targeting oxidative stress during peritoneal dialysis may help preserve cellular function and reduce mortality in PD patients [3]. The use of agents protecting against oxidative damage is not new [40,41]. Targeting the thioredoxin system or applying antioxidant compounds is of considerable interest for treating various diseases [42,43,44,45,46]. Consistent with our results are animal studies using Wistar rats receiving NAC, which significantly reduced functional and histological peritoneal changes, and which is encouraging for translation to patients [47]. Recently, it was shown that blocking ROS production could protect the peritoneal membrane [48,49]. Studies on albino rats treated with NAC, investigating the effect of NAC supplementation on the progression, as well as regression of encapsulating peritoneal sclerosis (EPS), demonstrated decreased inflammation and vascularity with NAC [50]. Noh and colleagues demonstrated that NAC supplementation prevented peritoneal fibrosis and membrane hyperpermeability in rats [51]. Previous in vitro and animal studies have suggested that NAC and other antioxidant interventions may attenuate oxidative stress-associated peritoneal alterations. These findings provide a rationale for further investigating ROS-targeting strategies in clinically relevant models. However, the clinical relevance and therapeutic potential of NAC in peritoneal dialysis patients remain to be established, as available clinical evidence is currently limited and heterogeneous.

A clinical study in peritoneal dialysis patients receiving NAC for several weeks demonstrated that NAC intervention had no side effects and reduced IL-6 levels compared to the placebo group. However, no change in OS markers could be observed [52]. A meta-analysis by Ye et al. concluded that NAC supplementation is generally safe in CKD patients and may confer potential benefits in terms of kidney function, inflammatory markers, and cardiovascular risk profiles, although further large-scale studies are warranted to confirm these findings [53]. A retrospective study by Liao and colleagues demonstrated that NAC treatment is associated with a reduced risk for progression to end-stage renal disease (ESRD) requiring dialysis [54]. Promising results are shown in the retrospective study by Chiu et al., demonstrating that the incidence of progression to hemodialysis was significantly lower among NAC users, possibly associated with more favorable trends in serum creatinine and eGFR compared to non-users [55]. By contrast, a small placebo-controlled trial, involving 20 non-diabetic patients with proteinuria and mildly reduced renal function, found that the combination therapy of NAC and renin–angiotensin–aldosterone system (RAAS) blockade had no significant impacts on proteinuria or surrogate markers of tubular injury and renal fibrosis [56]. As shown by several in vitro and animal studies, scavenging intracellular oxidative stress might be a potential strategy to maintain peritoneal membrane integrity.

### Limitations

Several limitations should be acknowledged. First, the experiments were performed using primary HPMCs derived from a limited number of donors (n = 3 for functional read-outs), which may constrain the generalizability of the findings. Future studies involving larger and clinically characterized donor cohorts will be required to further validate these observations and better define inter-individual variability in HPMC responses to oxidative stress and antioxidant intervention. Second, considering the median age of 67 years and the presence of diabetes in 20% of donors it cannot be excluded that these factors had already influenced TXNIP expression and consequently contributed to its increased expression levels [8,21,57]. Third, a high proportion of donors had arterial hypertension (90%), a condition known to be associated with chronic endothelial oxidative stress and dysfunction [58,59]. Therefore, it cannot be excluded that donor comorbidities influenced the baseline redox status of the isolated endothelial cells and potentially affected their response to the experimental interventions. However, hypertension is highly prevalent among patients with CKD, affecting approximately 60–90% of patients and up to 90% of those with advanced disease. Therefore, the high prevalence of hypertension in our donor cohort reflects the typical clinical characteristics of the CKD population rather than a selection bias. A fourth limitation is that cells were exposed to high glucose for only 3 h; this acute setting does not reproduce the chronic, long-term exposure that drives peritoneal membrane damage in vivo. A further limitation is the lack of molecular-level validation of Trx and TXNIP expression levels. Future studies using a larger and independent cohort with sufficient biological material should include comprehensive validation of Trx and TXNIP at both the mRNA and protein levels to confirm the observed regulatory changes and provide deeper mechanistic insights into the Trx/TXNIP pathway. Furthermore, the high glucose-induced reduction in cell viability may have partially confounded the fluorescence-based quantification of TXNIP, intracellular oxidative stress, and γH2Ax. In addition, antioxidant enzyme regulation was assessed only at the mRNA level, without confirmation at the protein or functional activity level. Therefore, prospective studies and in vivo investigations are warranted to validate these findings and further evaluate the therapeutic potential of antioxidant strategies. NAC was used as a well-characterized pharmacological modulator of cellular redox homeostasis to examine the contribution of intracellular oxidative stress to high glucose-induced DNA damage. Together with the CellROX Green measurements demonstrating increased intracellular oxidative stress under high-glucose conditions and its attenuation by NAC, these findings support the involvement of oxidative stress in the observed phenotype. However, because NAC exerts multiple biological effects beyond reducing oxidative stress, our data should be interpreted as evidence for the contribution of oxidative stress rather than as proof that ROS are the exclusive mediator or as support for NAC as a clinically therapeutic agent.

## 5. Conclusions

**Figure 6:**
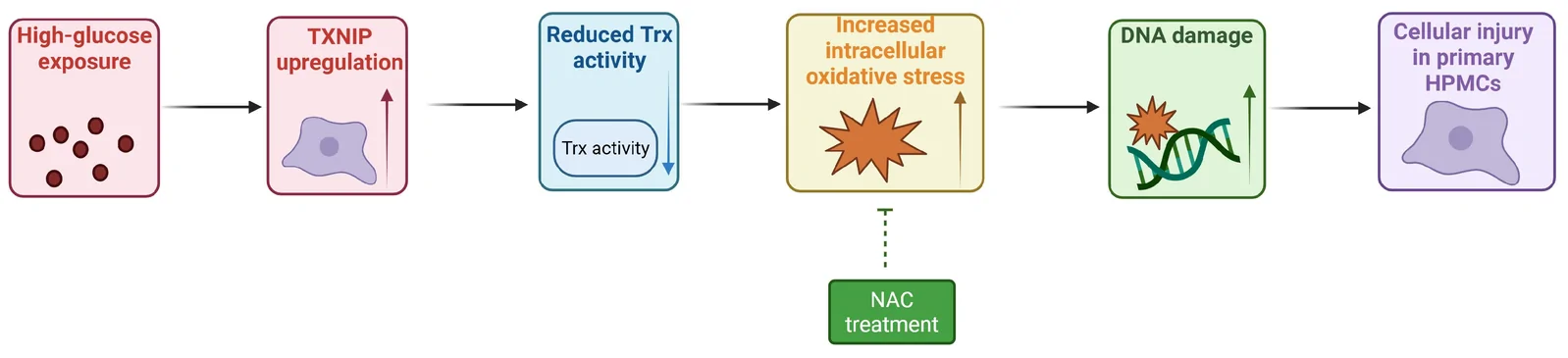
Proposed mechanism underlying high-glucose-induced cellular injury in primary HPMCs. High-glucose exposure induces TXNIP upregulation, resulting in reduced Trx activity and consequently increased intracellular oxidative stress. Elevated oxidative stress promotes DNA damage and ultimately contributes to cellular injury in primary HPMCs. NAC treatment attenuates intracellular oxidative stress, supporting a role for oxidative stress as a key mediator of high-glucose-induced cellular injury. Colored arrows indicate upregulation or downregulation relative to the box/compartment in which the arrow is located. Arrow direction indicates direction of regulation. Dashed line indicate a pharmacological intervention. TXNIP: Thioredoxin-interacting protein, Trx: Thioredoxin, HPMCs: human peritoneal mesothelial cells, NAC: N-acetylcysteine. Created with BioRender.com.

In conclusion, high-glucose exposure is associated with TXNIP upregulation, reduced thioredoxin activity, increased intracellular oxidative stress, and DNA damage in primary HPMCs. The attenuation of oxidative stress-associated signals and DNA damage following NAC treatment is consistent with an involvement of oxidative stress in the cellular response to high-glucose exposure. Given the pleiotropic effects of NAC, these findings do not establish a definitive ROS-mediated mechanism, but support the contribution of altered redox homeostasis to glucose-induced cellular injury (Figure 6). These findings provide further insights into redox dysregulation in HPMC injury and provide a basis for further investigation of oxidative stress and redox dysregulation in HPMCs injury. Further in vivo and translational studies are required to determine the clinical relevance of these observations.

## Figures and Tables

**Table 1:** Clinical data of study patients.

Variable	
Number	10
Female/male (*n*)	4/6
Age [years], median (IQR)	67 (55.5–68.5)
BMI [kg/m^2^], median (IQR)	25.6 (21.8–32.3)
Diabetes, *n* (%)	2 (20)
Hypertension, *n* (%)	9 (90)
Non smoker, *n* (%)	6 (60)
Active smoker, *n* (%)	2 (20)
Former smoker, *n* (%)	2 (20)
Laboratory	
Leucocytes [GIGA/L], median (IQR)	6.8 (5.6–7.7)
Hemoglobin [g/L], median (IQR)	105.0 (82.5–109.3)
C-reactive protein (CRP) [mg/dL], median (IQR)	0.95 (0.18–1.68)
Creatinine [mg/dL], median (IQR)	6.57 (5.64–10.03)
Glomerular Filtration Rate GFR (CKD-EPI [mL/min/1.73 m^2^], median (IQR)	7.5 (3.75–9.0)
Urea [mg/dL], median (IQR)	169.0 (122.3–213.5)

*n*: number of values, IQR: interquartile range, kg: kilogram, m^2^: square of the body height, BMI: body mass index.

## Data Availability

The datasets used and/or analyzed during the current study are available from the corresponding author on reasonable request.

## Supplementary Materials

The following supporting information can be downloaded at: https://www.mdpi.com/article/10.3390/antiox15081032/s1, Figure S1. Verification of human peritoneal mesothelial cells (HPMCs). Representative confocal microscopy images of human peritoneal mesothelial cells isolated from dialysis effluent, stained for calretinin (red) and vimentin (green). Nuclei were counterstained with DAPI (blue). Scale bar: 25 μm; Figure S2. Analysis of TXNIP and TRX mRNA expression levels following high-glucose exposure. TXNIP and TRX mRNA expression levels were assessed by quantitative real-time PCR in primary HPMCs from one representative patient following high-glucose treatment. Data are presented as relative mRNA expression normalized to the respective control condition. TXNIP: Thioredoxin-interacting protein, TRX: Thioredoxin.

## Abbreviations

The following abbreviations are used in this manuscript:

HPMCs	Human peritoneal mesothelial cells
DNA	Deoxyribonucleic acid
PD	Peritoneal dialysis
Trx	Thioredoxin
TXNIP	Thioredoxin-interacting protein
ROS	Reactive oxygen species
NAC	N-Acetylcysteine
RRT	Renal replacement therapy
PDF	Peritoneal dialysis fluid
OS	Oxidative stress
MT	Metallothionein
SOD	Superoxide dismutase
CAT	Catalase
GPx	Glutathione peroxidase
PDE	Peritoneal dialysis effluent
BMI	Body mass index
MFI	Mean fluorescence intensity
PM	Peritoneal membrane
CKD	Chronic kidney disease
IL	Interleukin
ESRD	End-stage renal disease
