# Supplementary material for: N-Acetylcysteine Protects HPMCs from High-Glucose-Induced Oxidative DNA Damage
Source: Antioxidants (Basel). 2026 Aug 19;15(8):1032. doi: 10.3390/antiox15081032 (PMC13509520; doi:10.3390/antiox15081032)
Supplement: Supplementary file 1 [file antioxidants-15-01032-s001.zip › antioxidants-4436760-supplementary.pdf]

Supplementary Materials

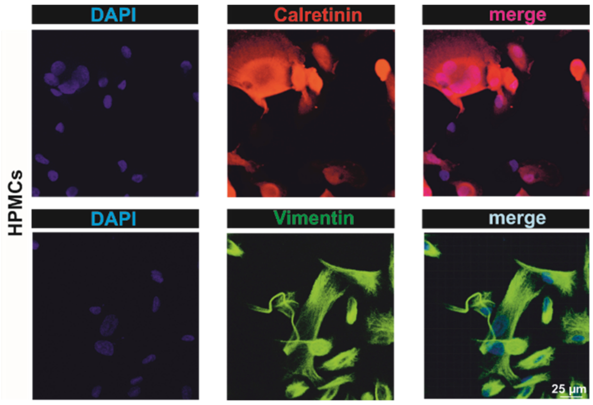

Figure S1. **Verification of human peritoneal mesothelial cells (HPMCs).**

Representative confocal microscopy images of human peritoneal mesothelial cells isolated from dialysis effluent, stained for calretinin (red) and vimentin (green). Nuclei were counterstained with DAPI (blue). Scale bar: 25 µm.

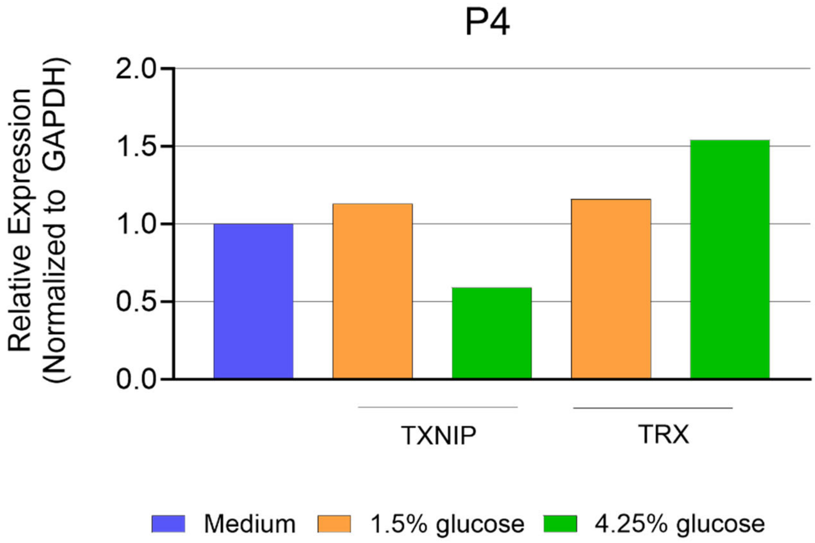

Figure S2. Analysis of TXNIP and TRX mRNA expression levels following high-glucose exposure.

TXNIP and TRX mRNA expression levels were assessed by quantitative real-time PCR in primary HPMCs from one representative patient following high-glucose treatment. Data are presented as relative mRNA expression normalized to the respective control condition. TXNIP: Thioredoxin-interacting protein, TRX: Thioredoxin
